# Hypoxia regulates TRAIL sensitivity of colorectal cancer cells through mitochondrial autophagy

**DOI:** 10.18632/oncotarget.9206

**Published:** 2016-05-06

**Authors:** Gertrud Knoll, Sebastian Bittner, Maria Kurz, Jonathan Jantsch, Martin Ehrenschwender

**Affiliations:** ^1^ Institute of Clinical Microbiology and Hygiene, University Hospital Regensburg, 93053 Regensburg, Germany

**Keywords:** TRAIL, SMAC mimetic, death receptor, hypoxia

## Abstract

The capacity of tumor necrosis factor-related apoptosis-inducing ligand (TRAIL) to selectively induce cell death in malignant cells triggered numerous attempts for therapeutic exploitation. In clinical trials, however, TRAIL did not live up to the expectations, as tumors exhibit high rates of TRAIL resistance *in vivo*. Response to anti-cancer therapy is determined not only by cancer cell intrinsic factors (e.g. oncogenic mutations), but also modulated by extrinsic factors such as the hypoxic tumor microenvironment.

Here, we address the effect of hypoxia on pro-apoptotic TRAIL signaling in colorectal cancer cells. We show that oxygen levels modulate susceptibility to TRAIL-induced cell death, which is severely impaired under hypoxia (0.5% O_2_). Mechanistically, this is attributable to hypoxia-induced mitochondrial autophagy. Loss of mitochondria under hypoxia restricts the availability of mitochondria-derived pro-apoptotic molecules such as second mitochondria-derived activator of caspase (SMAC), thereby disrupting amplification of the apoptotic signal emanating from the TRAIL death receptors and efficiently blocking cell death in type-II cells. Moreover, we identify strategies to overcome TRAIL resistance in low oxygen environments. Counteracting hypoxia-induced loss of endogenous SMAC by exogenous substitution of SMAC mimetics fully restores TRAIL sensitivity in colorectal cancer cells. Alternatively, enforcing a mitochondria-independent type-I mode of cell death by targeting the type-II phenotype gatekeeper X-linked inhibitor of apoptosis protein (XIAP) is equally effective.

Together, our results indicate that tumor hypoxia impairs TRAIL efficacy but this limitation can be overcome by combining TRAIL with SMAC mimetics or XIAP-targeting drugs. Our findings may help to exploit the potential of TRAIL in cancer therapy.

## INTRODUCTION

Among the tumor necrosis factor (TNF) ligands, TNF-related apoptosis-inducing ligand (TRAIL) is unique due to its capacity to selectively induce cell death in tumor cells [1]. TRAIL binding to TRAIL receptor 1 (TRAIL-R1) or TRAIL-R2 triggers assembly of a death-inducing signaling complex (DISC). This platform allows stepwise caspase-8 activation and initiates a cascade of proteolytic cleavage events that culminate in caspase-3 activation, finally triggering the execution phase of apoptosis. In so-called type-I cells, caspase-8-mediated cleavage of caspase-3 is followed by robust autocatalytic caspase-3 processing and efficient cell death induction. In type-II cells, however, X-linked inhibitor of apoptosis protein (XIAP) stalls caspase-3 maturation an intermediate step and has to be overridden by a mitochondria-derived amplification of the death signal. In this scenario, caspase-8 cleavage of the BH3-interacting domain death agonist (Bid) to tBid [2] activates Bcl2-associated X protein (Bax) and Bcl2-antagonist/killer (Bak). Bax and Bak form a pore complex in the outer mitochondrial membrane and unleash pro-apoptotic factors such as cytochrome c, HtrA2 and second mitochondria-derived activator of caspase (SMAC) [3]. Together with apoptotic-protease-activating factor 1 (Apaf-1), cytochrome c forms the ‘apoptosome’, a molecular scaffold for caspase-9 activation, which in turn boosts downstream effector caspase activation. SMAC and HtrA2 act synergistically by neutralizing cytosolic inhibitors of apoptosis proteins (IAPs), especially XIAP [4, 5].

Soon after its discovery, TRAIL emerged as a promising anti-cancer agent with encouraging results in pre-clinical studies. Disappointingly, the therapeutic benefit of TRAIL in clinical trials is to date rather limited [6]. The reasons for TRAIL's sobering clinical performance are incompletely understood. We and others previously showed that cancer-cell intrinsic factors, such as the oncogenic *PIK3CA* H1047R mutation conferred high-level TRAIL resistance in colorectal cancer cells [7, 8]. Additionally, extrinsic factors, such as the tumor microenvironment affect the response to anti-cancer therapies [9]. In solid malignancies, fast proliferation can outgrow the supply of nutrients and oxygen provided by the malformed tumor vasculature. Hypoxia is common in human cancers and grants stabilization of hypoxia-inducible factor 1α (HIF1α). This transcription factor ensures cell survival through adaptive changes in cell metabolism (reviewed in [10]). Notably, HIF1α has also been implicated in carcinogenesis and metastasis of colorectal cancer [11] and overexpression is associated with poor prognosis [12].

Here, we show that hypoxia induced TRAIL resistance in colorectal cancer cells. Oxygen deprivation reduced the levels of mitochondria-derived pro-apoptotic SMAC and HtrA2 molecules by hypoxia-induced mitophagy, thereby disrupting mitochondria-dependent amplification of the TRAIL-triggered death signal and blocking apoptosis in type-II cells. Inhibition of hypoxia-induced mitophagy or replacement of endogenous SMAC with exogenously added SMAC mimetics fully restored TRAIL cytotoxicity under hypoxic conditions. Additionally, switching type-II cells to a type-I mode of cell death by targeting the type-II phenotype gatekeeper XIAP rendered mitochondrial death signal amplification dispensable and allowed full-blown TRAIL-induced apoptosis under hypoxic conditions. Together, we identified hypoxia as an extrinsic modulator of TRAIL susceptibility in colorectal cancer cells. Therapeutically, our results indicate that combinatorial treatments with TRAIL and SMAC mimetics or XIAP-targeting drugs can overcome hypoxia-induced TRAIL resistance and may offer a promising strategy to exploit the potential of TRAIL in cancer therapy.

## RESULTS

### Hypoxia reduces TRAIL-induced cell death in colorectal cancer cells

**Figure 1:**
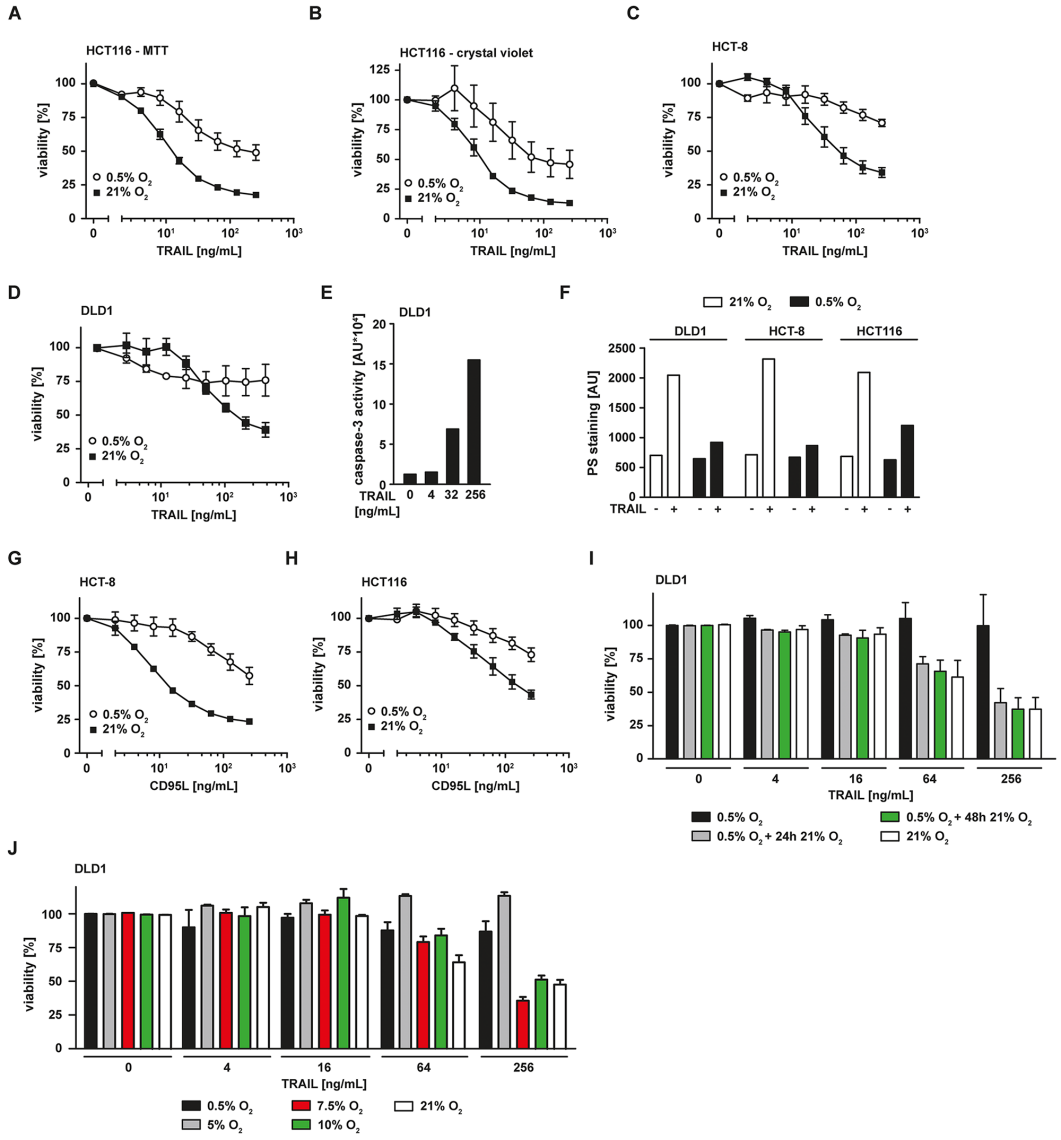
Hypoxia reduces TRAIL-induced cell death in colorectal cancer cells **A–D.** HCT116, HCT-8 and DLD1 cells were grown under normoxic (21% O_2_) or hypoxic (0.5% O_2_) conditions for 18 h. Subsequently, cells were challenged with the indicated concentrations of TRAIL for another 18 h. Viability was measured using MTT (A, C, D) or crystal violet (B) staining. Values are means ± SEM from three experiments. **E.** DLD1 cells were challenged with the indicated concentrations of TRAIL for 5 h. Caspase-3 activity was measured using the fluorogenic substrate (DEVD)_2_-R110. One representative experiment of three performed is shown. AU, arbitrary units. **F.** DLD1, HCT-8 and HCT116 cells were grown under normoxic (21% O_2_) or hypoxic (0.5% O_2_) conditions for 18 h. Subsequently, cells were challenged with 256 ng/mL TRAIL for 3 h or left untreated. Translocation of phosphatidylserine to the outer leaflet of the plasma membrane was measured using a fluorogenic Annexin V derivate. One representative experiment of two performed is shown. PS, phosphatidylserine. **G, H.** HCT-8 and HCT116 cells were grown under normoxic (21% O_2_) or hypoxic (0.5% O_2_) conditions for 18 h. Subsequently, cells were challenged with the indicated concentrations of CD95L for additional 18 h. Cell viability was measured using MTT staining. Values are means ± SEM from three experiments. **I.** DLD1 cells were grown under normoxic (21% O_2_) or hypoxic (0.5% O_2_) conditions for 18 h. Hypoxia-experienced cells were subsequently split in three groups and grown in an environment containing 0.5% O_2_ (black bars) for 24 h or 21% O_2_ for 24 h (grey bars) or 48 h (green bars). Subsequently, cells were challenged with the indicated concentrations of TRAIL for 18 h. Cell viability was measured using MTT staining. Values are means ± SEM from three experiments. **J.** DLD1 cells were grown under normoxic (21% O_2_) or various hypoxic (0.5% - 10% O_2_) conditions for 18 h. Subsequently, cells were challenged with the indicated concentrations of TRAIL for another 18 h. Viability was measured using MTT staining. Values are means ± SEM from three experiments.

Hypoxia (0.5% O_2_) significantly attenuated TRAIL-induced cell death in the colorectal cancer cell lines HCT116 (Figure 1A), HCT-8 (Figure 1C) and DLD1 (Figure 1D) compared to normoxia (ambient air, ~21% O_2_) in MTT- (Figure 1A, 1C, 1D) and crystal violet-based viability assays (Figure 1B). Expectedly, TRAIL-induced loss of viability under normoxic conditions was associated with activation of caspase-3, a prototypic effector caspase in apoptosis (Figure 1E). TRAIL-triggered translocation of phosphatidylserine (PS) to the outer leaflet of the plasma membrane, another hallmark of ongoing apoptosis, was prominent under normoxia but tremendously reduced under hypoxia (Figure 1F). We next investigated whether hypoxia selectively impairs TRAIL death receptor-mediated cytotoxic effects or also influences pro-apoptotic signaling of other death receptors such as CD95. Indeed, hypoxia attenuated cell death in CD95L-treated HCT-8 (Figure 1G) and HCT116 cells (Figure 1H), thereby pointing to a more general role of oxygen levels in modulating death receptor-associated pro-apoptotic signaling pathways. Hypoxia-mediated TRAIL resistance was dependent on the persistent absence of oxygen and rapidly vanished when normoxic conditions were restored (Figure 1I). The attenuation of TRAIL-induced cell death visible in DLD1 cells under hypoxic conditions (black bars) was completely reversible by normoxic cultivation for additional 24 h (grey bars) or 48 h (green bars) before adding TRAIL. Additionally, the extent of hypoxia-induced TRAIL resistance correlated with the levels of available oxygen (Figure 1J). Whereas TRAIL-induced cell death was strongly inhibited in the presence of 0.5% O_2_ (black bars) and 5% O_2_ (grey bars), oxygen levels of 7.5% (red bars) and above fully restored TRAIL cytotoxicity to normoxic levels (white bars). Notably, oxygen levels between 5 and 10% are physiologically encountered in various tissues [13]. Together, these date demonstrated that oxygen levels modulate death receptor-induced cell death in colorectal cancer cells.

### Hypoxia alters expression of pro- and anti-apoptotic proteins

**Figure 2:**
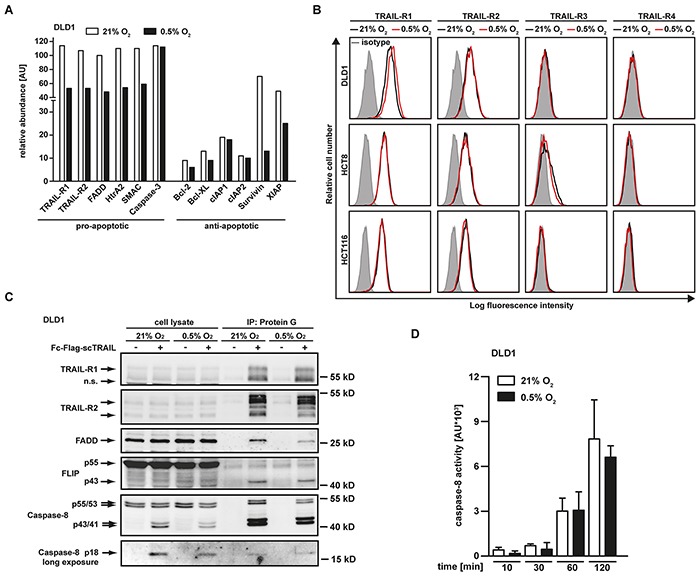
Hypoxia changes the expression levels of pro- and anti-apoptotic proteins but does not impair TRAIL-induced DISC formation **A.** DLD1 cells were grown under normoxic (21% O_2_) or hypoxic (0.5% O_2_) conditions for 18 h. Subsequently, cells were lysed and relative expression levels of the indicated proteins were measured using an antibody-based array (see Material and Methods section). Proteins were quantified using densitometry and normalized to reference spots. Data shown are representative of two experiments performed. AU, arbitrary units. **B.** DLD-1, HCT-8 and HCT116 cells were grown for 18 h under normoxic (21% O_2_) or hypoxic (0.5% O_2_) conditions and subsequently analyzed for TRAIL-R1, TRAIL-R2, TRAIL-R3 and TRAIL-R4 cell surface expression using flow cytometry. Data shown are representative of three experiments performed. **C.** TRAIL-R1/−R2 signaling complexes were induced in DLD1 cells grown for 18 h under normoxic (21% O_2_) or hypoxic (0.5% O_2_) conditions by stimulation with Fc-FLAG-scTRAIL (1 μg/mL) for 90 min. Proteins associated with Fc-FLAG-scTRAIL were immunoprecipitated using protein G agarose and were analyzed together with the corresponding lysates by Western blotting for the presence of TRAIL-R1 and TRAIL-R2 and the DISC components FADD, FLIP and caspase-8. Data shown are representative of two experiments performed. **D.** DLD1 cells were grown for 18 h under normoxic (21% O_2_) or hypoxic (0.5% O_2_) conditions and subsequently challenged with 256 ng/mL TRAIL for the indicated periods of time. Caspase-8 activity was measured using the fluorogenic substrate (Ac-IETD)_2_-R110. Values are means ± SEM from three experiments. AU, arbitrary units.

We next addressed the molecular mechanisms underlying hypoxia-induced TRAIL resistance in colorectal cancer cells. Using an antibody-based protein array (Figure 2A), we measured oxygen-dependent changes in the abundance of pro- and anti-apoptotic proteins in cell lysates. Hypoxia not only reduced expression levels of TRAIL-R1, TRAIL-R2 and Fas-associated death domain (FADD), but also lowered the abundance of mitochondria-derived pro-apoptotic factors such as HtrA2 and SMAC. Expression of caspase-3, an essential molecule in the effector phase of apoptosis, did not significantly differ in hypoxic and normoxic conditions (Figure 2A). Therefore, hypoxia most likely blocked activation of the effector phase of apoptosis rather than abolishing the effector mechanism itself. Oxygen deprivation also reduced the levels of the anti-apoptotic proteins Survivin and XIAP, whereas the levels of Bcl-2, Bcl-X_L_, cIAP1 and cIAP2 were only minimally affected. Apparently, this reduction was not sufficient to enhance TRAIL sensitivity, as hypoxia decreased rather than increased TRAIL-induced cytotoxicity in colorectal cancer cells (Figure 1). To validate our proteome array data, we examined whether the hypoxia-induced loss of pro-apoptotic proteins functionally contributes to the attenuated TRAIL sensitivity observed in a low oxygen environment. Importantly, a mere reduction of a pro-apoptotic molecule in cell lysates not necessarily promotes survival, as factor-specific thresholds and the subcellular location of proteins have to be considered. For example, the observed decrease in the total cellular amount of TRAIL-R1 and TRAIL-R2 under hypoxia allows no conclusion regarding the cell surface expression of the TRAIL death receptors. Ligand binding to death receptors occurs at the plasma membrane and initiates the apoptosis-inducing signaling cascade, cell surface localization is therefore functionally of outstanding importance (for review see [14]). Flow cytometry revealed that the observed reduction in the total amount of TRAIL-R1 and TRAIL-R2 under hypoxic conditions did not correlate with decreased expression of these death receptors at the cell surface (Figure 2B). TRAIL-R1 and TRAIL-R2 surface expression under hypoxia was comparable to normoxia. Additionally, lowering oxygen levels to 0.5% did not affect expression levels of the decoy receptors TRAIL-R3 and TRAIL-R4. In line with these findings, immunoprecipitation experiments demonstrated that TRAIL-induced receptor signaling complex formation occurred equally effective under hypoxic or normoxic conditions (Figure 2C). Notably, we did not detect significant oxygen-dependent differences in the amount of precipitated TRAIL-R1 and TRAIL-R2. Although total cellular FADD levels were reduced under hypoxia (Figure 2A), FADD was precipitated together with TRAIL-R1 and TRAIL-R2 (Figure 2C), suggesting that the remaining amount was still sufficient to form a functional death receptor-associated signaling complex. In line with this, TRAIL-induced processing of the caspase-8 p55/53 pro-form via the p43/41 intermediate at the DISC to the enzymatically active p18 fragment occurred roughly to the same extend under hypoxic or normoxic conditions (Figure 2C). Undisturbed TRAIL-induced caspase-8 activation under oxygen deprivation was also confirmed in caspase-8 activity assays (Figure 2D). Together, our data indicated that the initial steps of apoptosis induction were intact and hypoxia blocked TRAIL-induced cell death downstream of receptor-ligation, DISC formation and caspase-8 activation.

### Hypoxia induces loss of mitochondrial mass

**Figure 3:**
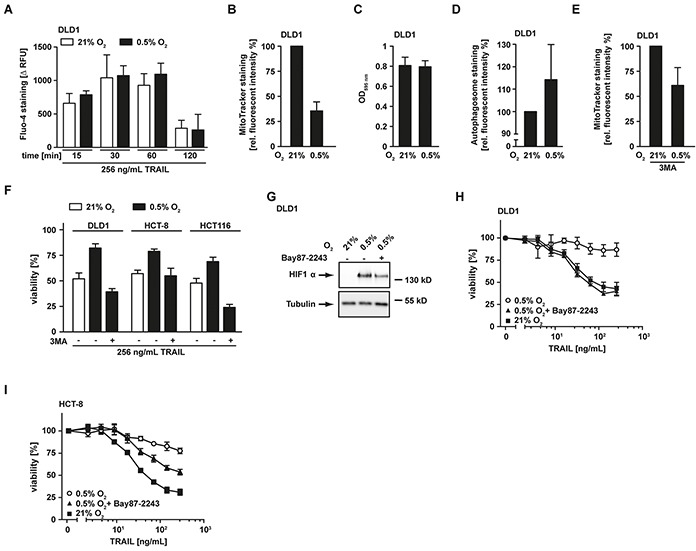
Hypoxia-induced mitochondrial autophagy decreases TRAIL sensitivity **A.** DLD1 cells were grown under normoxic (21% O_2_, white bars) and hypoxic (0.5% O_2_, black bars) conditions for 18 h and subsequently challenged with 256 ng/mL TRAIL for the indicated periods of time. Cytosolic Ca^2+^ levels were measured using Fluo-4 staining and quantified in a microplate reader. To calculate TRAIL-induced Ca^2+^ influx (Δ RFU), the fluorescence intensity from untreated controls was subtracted from every condition. Values are means ± SEM from three experiments. RFU, relative fluorescent units. **B.** DLD1 cells were grown as in (A) followed by staining of mitochondria using MitoTracker Green FM. Fluorescence intensity was quantified using a microplate reader and is given relative to the normoxic control. Values are means ± SEM from three experiments. **C.** DLD1 cells were grown as in (A) and cell proliferation was measured using MTT staining. Values are means ± SEM from three experiments. **D.** Cells were treated as in (A) followed by autophagosome staining. Fluorescence intensity was quantified using a microplate reader and is given relative to the normoxic control. Values are means ± SEM from three experiments. **E.** DLD1 cells were grown as in (A) in the presence of the autophagy inhibitor 3MA (12 mM). Subsequently, mitochondria were stained using MitoTracker and fluorescence was quantified in a microplate reader. Data are given relative to the corresponding normoxic controls. Values are means ± SEM from three experiments. **F.** DLD1, HCT-8 and HCT116 cells were grown as in (A) in the presence and absence of the autophagy inhibitor 3MA (12 mM) and subsequently challenged with the TRAIL (256 ng/mL) for 18 h. Viability was measured using MTT staining. Values are means ± SEM from three experiments. **G.** DLD1 cells were grown for 18 h under normoxic (21% O_2_) and hypoxic (0.5% O_2_) conditions in the presence and absence of the HIF1α inhibitor Bay87-2243 (50 μM). HIF1α levels were analyzed by Western blotting, tubulin served as loading control. **H, I.** DLD1 and HCT-8 cells were grown as in (A) in the presence and absence of the HIF1α inhibitor Bay87-2243 (25 μM) and subsequently challenged with the indicated concentrations of TRAIL for 18 h. Viability was measured using MTT staining. Values are means ± SEM from three experiments.

Following activation of initiator caspases at the DISC, release of mitochondria-derived apoptosis-promoting proteins such as HtrA2 and SMAC are in type-II cells essential (and in type-I cells accelerative) for full-blown effector caspase activation and cell death. The reduced HtrA2 and SMAC levels under low oxygen conditions (Figure 2A) could therefore impair TRAIL-induced cell death downstream of DISC formation. Efficient release of apoptotic proteins from the intermembrane space of mitochondria depends on a stimulus-triggered increase in cytosolic Ca^2+^ [15]. In CD95 signaling, CD95L-induced Ca^2+^ efflux from the endoplasmic reticulum is important for mitochondrial permeabilization and CD95-mediated apoptosis [16, 17]. We analyzed TRAIL-induced changes in cytosolic Ca^2+^ under hypoxic and normoxic conditions (Figure 3A). In both scenarios, TRAIL was comparably effective in augmenting cytosolic Ca^2+^, suggesting that oxygen-dependent differences in Ca^2+^ fluxes are unlikely to explain the lower levels of mitochondria-derived pro-apoptotic molecules under hypoxia.

Notably, a previous study demonstrated that hypoxia triggered mitochondrial autophagy as an adaptive metabolic response [18]. Staining of mitochondria in DLD1 cells grown under hypoxic conditions using the membrane potential-independent MitoTracker dye revealed a reduced staining intensity compared to normoxic controls (Figure 3B). This pointed to a hypoxia-induced decrease of mitochondrial mass. Importantly, cell proliferation was comparable under normoxic and hypoxic conditions (Figure 3C), excluding simple differences in cell number as causative for the decrease in mitochondrial mass under low oxygen. Additionally, enhanced autophagosome formation was detectable under hypoxic conditions (Figure 3D). In line with hypoxia-induced mitophagy, pharmacological blockade of autophagy using 3-methyladenine (3MA) attenuated the loss of mitochondria (Figure 3E). Moreover, 3MA overcame TRAIL resistance in hypoxic DLD1, HCT-8 and HCT116 cells (Figure 3F), thereby demonstrating the functional relevance of mitophagy for hypoxia-induced TRAIL resistance. In the absence of oxygen, HIF1α ensures (tumor) cell survival by coordinating the broad metabolic reprogramming at the transcriptional level (reviewed in [10]). DLD1 cells grown under hypoxic conditions expectedly stabilized HIF1α (Figure 3G). Interestingly, a recent study demonstrated a crucial role for HIF1α in hypoxia-induced mitophagy [18]. Consequently, inhibiting HIF1α in our experimental system should decrease hypoxia-induced mitophagy and enhance TRAIL-induced cell death. Indeed, blocking HIF1α using the pharmacological inhibitor Bay87-2243 [19] (Figure 3G) attenuated or even abolished oxygen-dependent differences in TRAIL sensitivity (Figures 3H and 3I). In sum, we demonstrated that oxygen deprivation reduced TRAIL sensitivity of colorectal cancer cells via hypoxia-induced mitophagy.

### SMAC mimetics overcome hypoxia-induced TRAIL resistance

**Figure 4:**
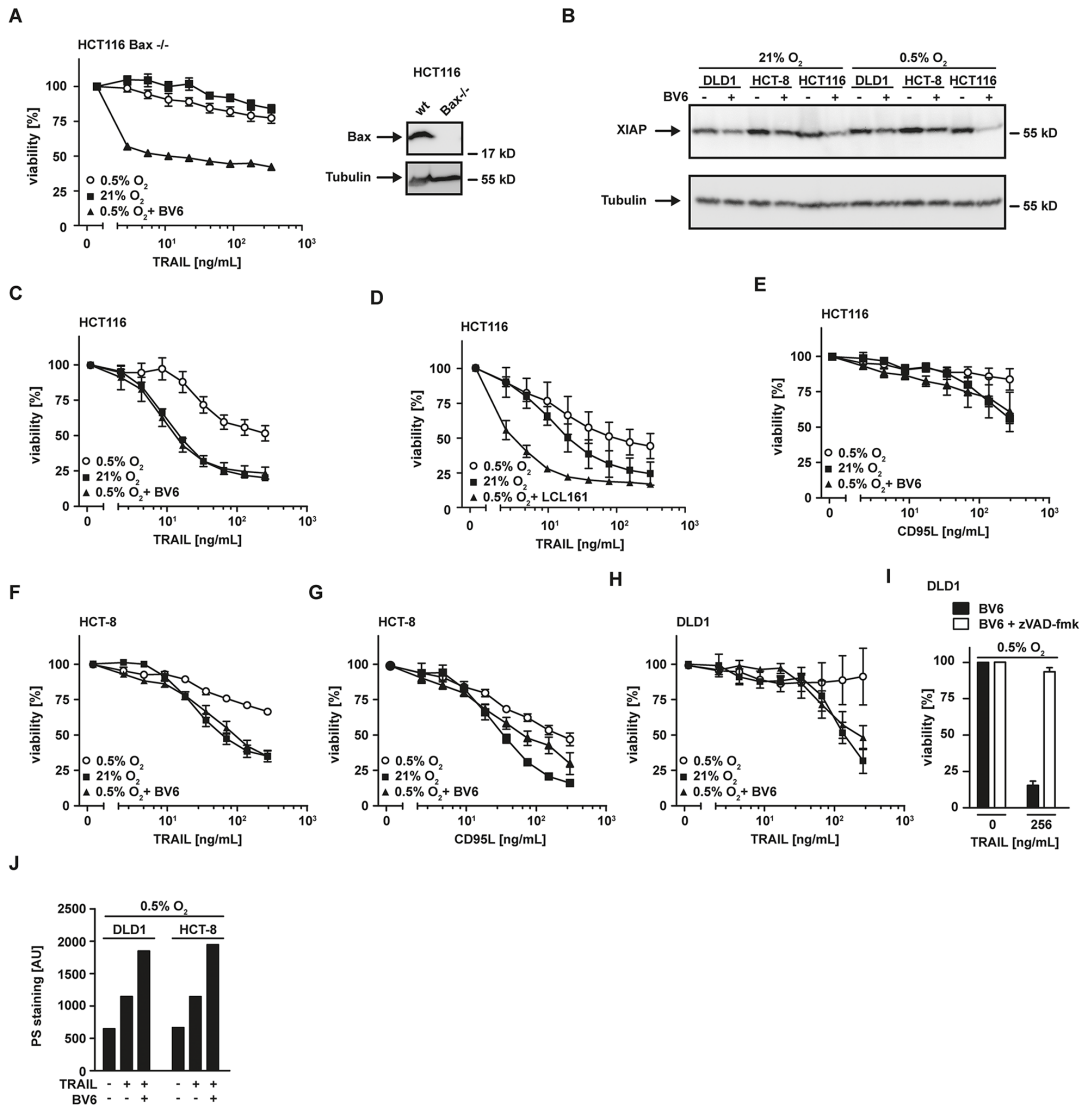
SMAC-mimetics overcome hypoxia-induced apoptosis resistance **A.** left panel: HCT116 Bax^−/−^ cells were grown under normoxic (21% O_2_) and hypoxic (0.5% O_2_) conditions for 18 h in the presence and absence of BV6 (6.25 μM). Subsequently, cells were challenged with the indicated concentrations of TRAIL for another 18 h. Viability was measured using MTT staining. Values are means ± SEM from three experiments. Right panel: Bax expression in HCT116 wildtype and HCT116 Bax^−/−^ cells was assessed by Western blotting. Tubulin served as loading control. **B.** HCT116, HCT-8 and DLD1 cells were grown as in (A) in the presence and absence of the SMAC mimetic BV6 (HCT116: 6.25 μM; HCT8: 6.25 μM; DLD1: 25 μM). XIAP levels were analyzed by Western blotting, tubulin served as loading control. **C–H.** HCT116, HCT-8 and DLD1 cells were grown as in (A) in the presence and absence of the SMAC mimetics BV6 (HCT116: 6.25 μM; HCT8: 6.25 μM; DLD1: 25 μM) or LCL161 (25 μM). Thereafter, cells were stimulated with the indicated concentrations of TRAIL or CD95L for 18 h and cell viability was measured using MTT staining. Values are means ± SEM from three experiments. **I.** DLD1 cells were grown under hypoxic conditions (0.5% O_2_) for 18 h in the presence of BV6 (25 μM, black bars) or BV6 + zVAD-fmk (100 μM, white bars). Subsequently, cells were treated with the indicated concentrations of TRAIL for 18 h. Cell viability was measured using MTT staining. Values are means ± SEM from three experiments. **J.** DLD1 and HCT-8 cells were grown under hypoxic (0.5% O_2_) conditions for 18 h in the presence and absence of BV6 (DLD1: 25 μM; HCT-8: 6.25 μM). Subsequently, cells were challenged with the indicated concentrations of TRAIL for 3 h. Translocation of phosphatidylserine to the outer leaflet of the plasma membrane was measured using a fluorogenic Annexin V derivate. One representative experiment of two performed is shown. PS, phosphatidylserine.

The observed loss of mitochondria-derived pro-apoptotic proteins (such as SMAC) under hypoxic conditions (Figure 2A) was accompanied by a decrease in mitochondrial mass (Figure 3B) and impaired TRAIL sensitivity, suggesting that the release of apoptosis-promoting factors from mitochondria is essential for efficient propagation of the apoptotic signal. In line with this, HCT116 cells deficient for Bax (HCT116 Bax^−/−^), a crucial player in mitochondrial permeabilization during apoptosis, exhibited high-level TRAIL resistance irrespective of the surrounding oxygen levels (Figure 4A). Consequently, restoring or imitating the mitochondria-mediated amplification of the apoptotic signal, e.g. with small-molecules mimicking the endogenous IAP antagonist SMAC (SMAC mimetics), could allow effective TRAIL-based treatments even in hypoxic tumor microenvironments. The SMAC mimetic BV6 efficiently reduced cellular levels of XIAP (Figure 4B) and re-sensitized HCT116 Bax^−/−^ (Figure 4A), HCT116, HCT-8 and DLD1 cells grown under hypoxic conditions to TRAIL- and/or CD95L-induced cell death (Figure 4C and 4E-4H). Notably, enhanced cytotoxicity of TRAIL in the presence of SMAC mimetics was not attributable to BV6-induced autocrine TNF secretion, as TNF was not detectable in the supernatant of BV6-treated cells (data not shown). LCL161, a SMAC mimetic that already entered phase II clinical trials (reviewed in [20]), also increased TRAIL-induced cell death under low oxygen levels (Figure 4D). The pan-caspase inhibitor zVAD-fmk completely abrogated TRAIL-induced cell death in BV6-treated DLD1 cells under hypoxia (Figure 4I) and combinatorial treatment with BV6 and TRAIL strongly increased PS exposure at the outer leaflet of the plasma membrane (Figure 4J), together pointing to ongoing apoptotic rather than necroptotic cell death. Collectively, our data highlighted that SMAC mimetics efficiently overcame hypoxia-induced TRAIL resistance in colorectal cancer cells.

### XIAP is critical for hypoxia-induced TRAIL resistance

**Figure 5:**
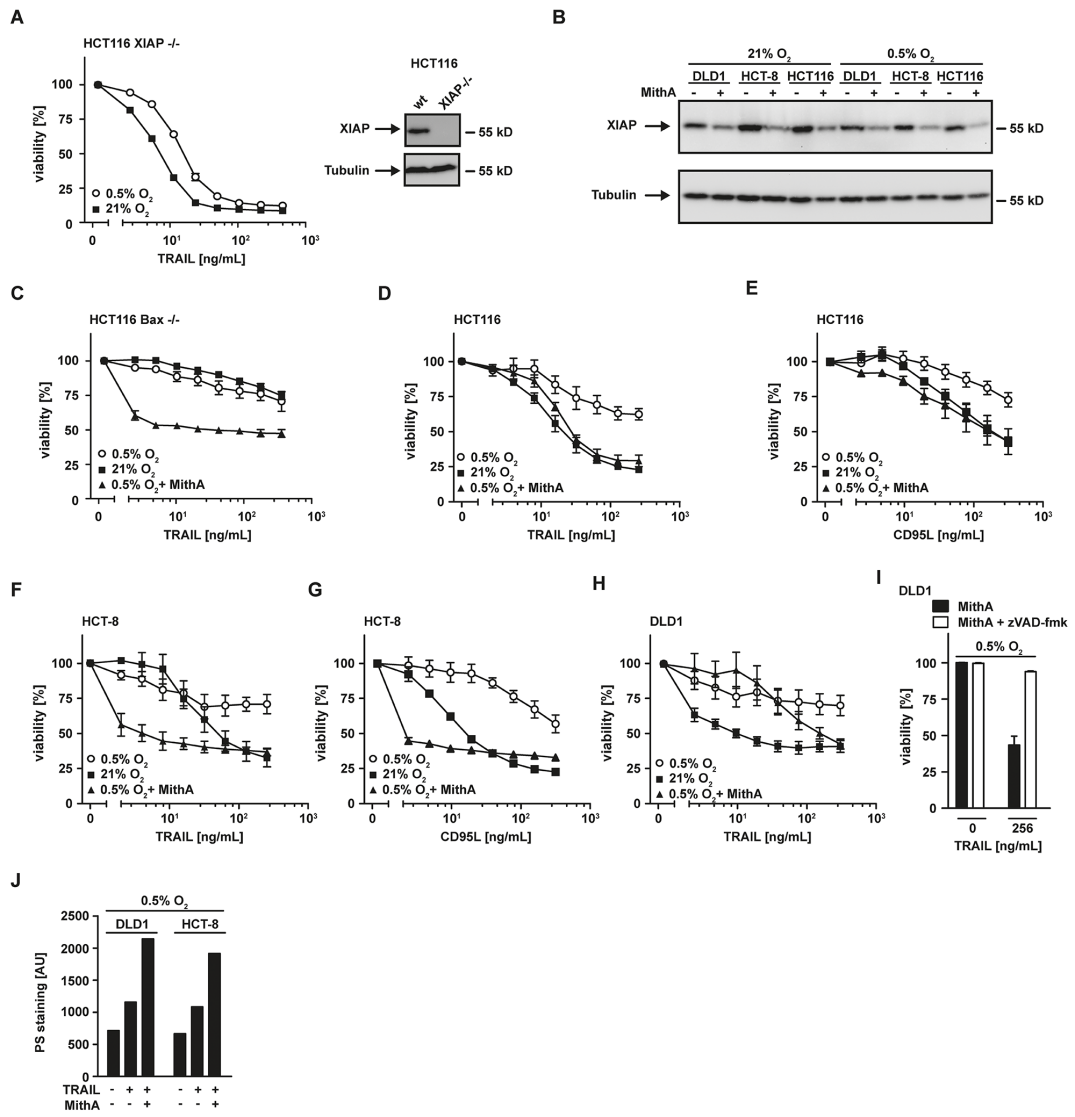
XIAP is critical for hypoxia-induced TRAIL resistance **A.** left panel: HCT116 XIAP^−/−^ cells were grown under normoxic (21% O_2_) and hypoxic (0.5% O_2_) conditions for 18 h. Following stimulation with the indicated concentrations of TRAIL for another 18 h, viability was measured using MTT staining. Values are means ± SEM from three experiments. Right panel: XIAP expression in HCT116 wildtype and HCT116 XIAP^−/−^ cells was assessed by Western blotting. Tubulin served as loading control. **B.** HCT116, HCT-8 and DLD1 cells were grown as in (A) in the presence and absence of mithramycin-A (HCT-8 and DLD1: 125 nM; HCT116: 10 nM). XIAP levels were analyzed by Western blotting, tubulin served as loading control. MithA, mithramycin-A. **C–H.** HCT116 Bax^−/−^, HCT116, HCT-8 and DLD1 cells were grown as in (A) in the presence and absence of mithramycin-A (HCT116 Bax^−/−^, HCT-8 and DLD1: 125 nM; HCT116: 10 nM) Thereafter, cells were stimulated with the indicated concentrations of TRAIL or CD95L for 18 h and cell viability was measured using MTT staining. Values are means ± SEM from three experiments. **I.** DLD1 cells were grown under hypoxic conditions (0.5% O_2_) for 18 h in the presence of MithA (125 nM, black bars) or MithA and zVAD-fmk (100 μM, white bars). Subsequently, cells were treated with the indicated concentrations of TRAIL for 18 h. Cell viability was measured using MTT staining. Values are means ± SEM from three experiments. **J.** DLD1 and HCT-8 cells were grown under hypoxic (0.5% O_2_) conditions for 18 h in the presence and absence of mithramycin-A (125 nM). Subsequently, cells were challenged with the indicated concentrations of TRAIL for 3 h. Translocation of phosphatidylserine to the outer leaflet of the plasma membrane was measured using a fluorogenic Annexin V derivate. One representative experiment of two performed is shown. PS, phosphatidylserine.

**Figure 6:**
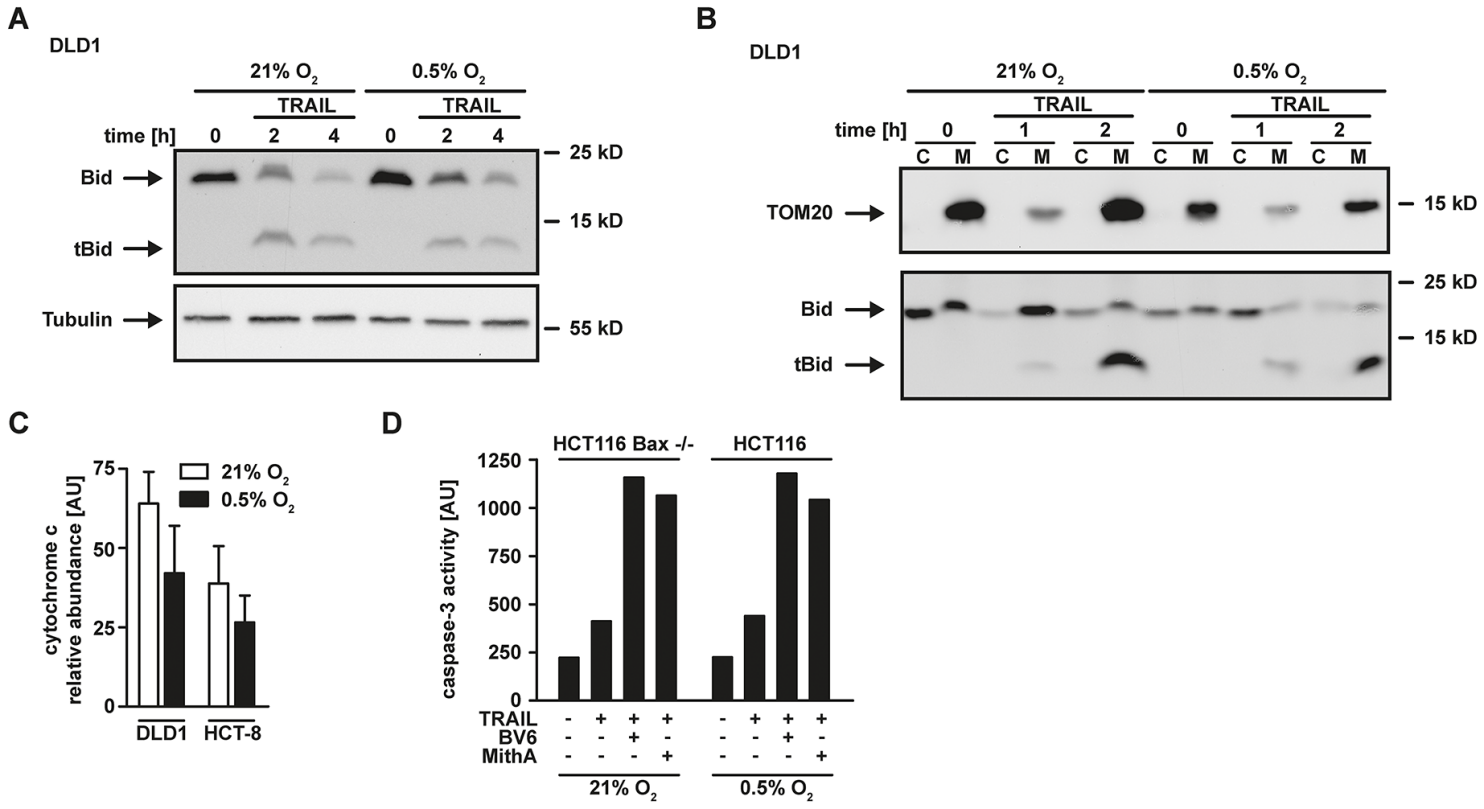
Hypoxia does not affect Bid cleavage and translocation to the mitochondria **A.** DLD1 cells were grown under normoxic (21% O_2_) and hypoxic (0.5% O_2_) conditions for 18 h. Subsequently, cells were challenged with 100 ng/mL TRAIL for the indicated periods of time. Processing of Bid into tBid was assessed by Western blotting. Tubulin served as loading control. **B.** Cells were grown as in (A) and challenged with 100 ng/mL TRAIL for the indicated periods of time. Subsequently, cells were harvested, lysed and separated into a cytosolic (“C”) and mitochondrial (“M”) fraction. Processing of Bid into tBid and mitochondrial translocation was assessed by Western blotting. Detection of TOM20 served as a mitochondrial marker protein to control sufficient fractionation of the lysates. **C.** DLD1 and HCT-8 cells were grown under normoxic (21% O_2_) or hypoxic (0.5% O_2_) conditions for 18 h. Subsequently, cells were lysed and relative expression levels of cytochrome c were measured using an antibody-based array (see Material and Methods section). Proteins were quantified using densitometry and normalized to reference spots. Data shown are means ± SEM from two experiments. AU, arbitrary units. **D.** In the presence and absence of BV6 (6.25 μM) or mithramycin-A (HCT116 Bax^−/−^: 125 nM, HCT116: 10 nM), HCT116 Bax^−/−^ were grown under normoxic (21% O_2_) conditions and HCT116 cells were grown under hypoxic conditions (0.5% O_2_) for 18 h. Subsequently, cells were challenged with TRAIL (128 ng/mL) for 4 h. Caspase-3 activity was measured using the fluorogenic substrate (DEVD)_2_-R110. One representative experiment of two performed is shown. AU, arbitrary units.

Apparently, hypoxia-induced mitophagy disrupted the required mitochondrial amplification of pro-apoptotic TRAIL signaling, thereby effectively blocking apoptosis in type-II cells. The anti-apoptotic protein XIAP acts as a gatekeeper of the type-II phenotype [21, 22]. We and others demonstrated that targeting XIAP is capable to convert type-II to type-I cells [21, 23, 24]. Our data indicated intact TRAIL-induced DISC formation under hypoxic conditions (Figure 2C). Consequently, enforcing a mitochondria-independent type-I mode of cell death should restore TRAIL cytotoxicity in hypoxia. In line with this hypothesis, XIAP-deficient HCT116 (HCT116 XIAP^−/−^) cells displayed a tremendously reduced TRAIL resistance under hypoxic conditions (Figure 5A) compared to HCT116 wildtype cells (Figure 1A). Blocking Sp1-mediated XIAP transcription using mithramycin-A (MithA) [25] (Figure 5B) enhanced TRAIL-induced cell death in HCT116 cells with a dysfunctional mitochondrial cell death pathway due to Bax-deficiency (Figure 5C). Moreover, MithA treatment of hypoxic HCT116 (Figures 5D and 5E), HCT-8 (Figures 5F and 5G) and DLD1 cells (Figure 5H) restored TRAIL- and CD95L-induced cell death. Inhibition of caspases using zVAD-fmk fully rescued MithA-treated DLD1 cells under hypoxia from TRAIL-induced cell death (Figure 5I) and combinatorial treatment with BV6 and TRAIL strongly increased PS exposure at the outer leaflet of the plasma membrane (Figure 5J). This argued for ongoing apoptosis in a mitochondria-independent type-I mode and we therefore next investigated the requirement for mitochondria-derived amplification of the death signal when XIAP is blocked. TRAIL-induced cleavage of the caspase-8 substrate Bid to tBid was equally effective in DLD1 cells grown under hypoxic or normoxic conditions (Figure 6A), which is in line with preserved DISC formation (Figure 2C) and caspase-8 activation (Figure 2D). In lysates from isolated mitochondria, we also observed no oxygen-dependent differences in TRAIL-induced Bid/tBid translocation to the mitochondria (Figure 6B), pointing to a resistance mechanism downstream of these initial steps of the mitochondrial amplification loop. Notably, hypoxia reduced the amount of the mitochondrial protein TOM20 (Figure 6B) and the total cellular content of cytochrome c (Figure 6C), which conforms to the previously observed hypoxia-induced loss of mitochondrial mass (Figure 3B). These observations support our hypothesis that hypoxia-induced mitophagy and concomitant reduction of mitochondria-derived pro-apoptotic molecules is the key mechanism for TRAIL-resistance granted during hypoxia. Restoring TRAIL-induced apoptosis using XIAP-targeting drugs did not depend on mitochondrial amplification of the death signal, as robust caspase-3 activation in HCT116 Bax^−/−^ cells was observable upon challenge with TRAIL+BV6 or TRAIL+MithA even though release of mitochondria-derived pro-apoptotic molecules was defective (Figure 6D). Likewise, hypoxia-induced mitophagy reduced TRAIL-triggered caspase-3 activation in HCT116 cells to levels of HCT116 Bax^−/−^ cells. Again, BV6 and MithA fully restored TRAIL-induced caspase-3 activation under hypoxic conditions. In sum, these data suggested that XIAP was essential for hypoxia-induced TRAIL resistance and targeting this druggable molecule rendered mitochondrial amplification of the apoptotic stimulus superfluous.

### Combinations of TRAIL with SMAC mimetics or mithramycin-A are also effective in colorectal cancer cells displaying apoptosis resistance under ample oxygen

**Figure 7:**
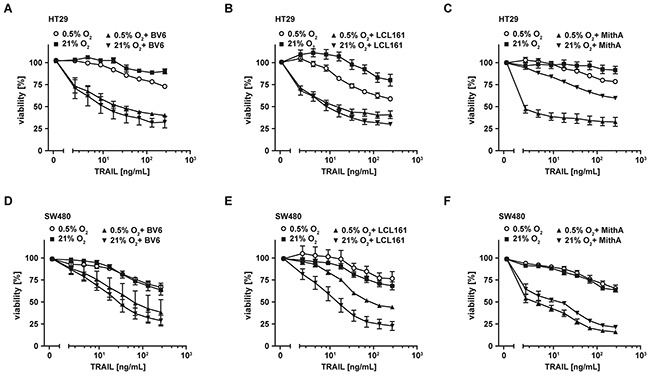
Combinations of TRAIL with SMAC mimetics or mithramycin-A also overcome TRAIL resistance under normoxic conditions **A–F.** HT29 and SW480 cells were grown under normoxic (21% O_2_) and hypoxic (0.5% O_2_) conditions for 18 h in the presence and absence of BV6 (6.25 μM) or LCL161 (HT29: 12.5 μM; SW480: 25 μM) or mithramycin-A (125 nM). Thereafter, cells were treated with the indicated concentrations of TRAIL for another 18 h. Cell viability was measured using MTT staining. Values are means ± SEM from three experiments. MithA, mithramycin-A.

Tumor cells may not depend on hypoxia to develop TRAIL resistance, but already exhibit this trait under normoxic conditions. Therefore, we questioned whether combinatorial treatment with TRAIL plus SMAC mimetics or MithA was also efficient in colorectal cancer cells with TRAIL resistance under normoxia. In combination with the SMAC mimetics BV6 or LCL161, TRAIL robustly induced cell death in HT29 (Figures 7A and 7B) and SW480 cells (Figures 7D and 7E). Comparable to SMAC mimetics, MithA also sensitized HT29 and SW480 cells to TRAIL-induced apoptosis (Figures 7C and 7F). Noteworthy, in the absence of these compounds the apoptotic response to TRAIL in both cell lines was at best moderate.

### Hypoxia reduces efficacy of apoptosis-inducing anti-cancer drugs in colorectal cancer cells

**Figure 8:**
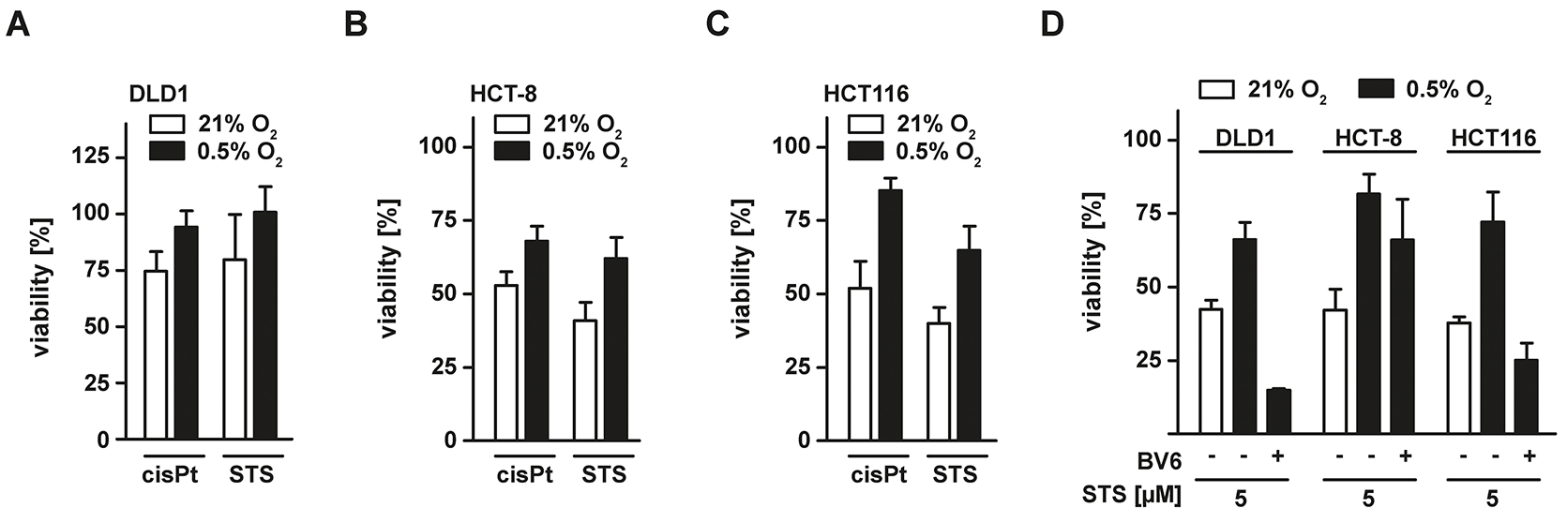
Hypoxia decreases sensitivity of colorectal cancer cells to apoptosis-inducing drugs **A–C.** DLD1, HCT-8 and HCT116 cells were grown under normoxic (21% O_2_) and hypoxic (0.5% O_2_) conditions for 18 h. Subsequently, cells were challenged with cis-platin (cisPt, 125 μM) or staurosporine (STS, 5 μM) for another 18 h. Cell viability was measured using MTT staining. Values are means ± SEM from three experiments. **D.** DLD1, HCT-8 and HCT116 were grown under normoxic (21% O_2_) and hypoxic (0.5% O_2_) conditions for 18 h in the presence and absence of BV6 (DLD1: 25 μM; HCT-8 and HCT116: 6.25 μM). Subsequently, cells were challenged with STS (5 μM) for another 18 h and cell viability was measured using MTT staining. Values are means ± SEM from three experiments.

We finally investigated whether apoptosis induction of anti-cancer drugs such as cis-platin (cisPt) and staurosporine (STS) was also impaired under low oxygen conditions. Compared to normoxia, we observed reduced cytotoxicity of both cisPt and STS in hypoxic DLD1, HCT-8 and HCT116 cells (Figure 8A–8C). Again, BV6 enhanced the cisPt- and STS-triggered cell death induction under low oxygen conditions (Figure 8D).

Summing up, our data demonstrate that colorectal cancer cells can acquire TRAIL resistance in a hypoxic environment. Mechanistically, this can be traced back to hypoxia-induced mitophagy. The later not only disables mitochondria-dependent amplification of death receptor-derived apoptotic signals (and thereby efficiently blocks cell death in type-II cells), but also impairs effectivity of other apoptosis-inducing anti-cancer drugs. Antagonizing anti-apoptotic IAP proteins using SMAC mimetics or enforcing a mitochondria-independent type-I mode of cell death by targeting XIAP restored sensitivity towards apoptosis regardless of oxygen levels and could also be exploited therapeutically.

## DISCUSSION

Hypoxia regulates numerous genes associated with tumor vascularization, invasion, drug resistance and metastasis (reviewed in [11]). The effects of hypoxia on TRAIL-induced cell death remain to date controversial. Initial observations indicated that hypoxia does not impair TRAIL-triggered cell death [26], but subsequent reports [27–30] and our current study found hypoxia-induced TRAIL resistance in a variety of cell lines (Figure 1). Notably, hypoxia not only increased resistance to TRAIL- but also to CD95L-, cisPt- and STS-induced cell death (Figure 1G and 1H, Figure 8). Oxygen deprivation may therefore act as a more general protective mechanism against apoptosis induction, possibly by disturbing an essential death promoting mechanism shared among different stimuli. In line with this, our data indicated that the hypoxia-induced cell death blockade is located downstream of death receptor activation and DISC formation, because cell surface expression of TRAIL-R1 and TRAIL-R2 (Figure 2B), recruitment of DISC components (Figure 2C) and caspase-8 activation (Figure 2D) were preserved under hypoxia. This is essentially in accordance with the previously reported intact TRAIL-induced caspase-8 and Bid cleavage in hypoxic HCT116 Bax^+/−^ cells, although that study did not examine death receptor expression and DISC formation [31].

The molecular mechanism that impairs TRAIL sensitivity in colorectal cancer cells under hypoxia is incompletely understood, but a pivotal role for mitochondria is emerging. Hypoxic conditions in TRAIL-treated HCT116 Bax^+/−^ reduced Bax translocation from the cytosol to the mitochondria, thereby impairing cytochrome c release, subsequent caspase-3 activation and apoptosis induction [28, 31]. However, these studies did not assess the effects of low oxygen levels on the cellular mitochondrial mass. Notably, hypoxia is a potent inducer of mitophagy [18] and a recent study elegantly demonstrated that the mitochondrial outer-membrane protein FUNDC1 links mitochondria to the autophagic machinery [32]. This study also reported reduced cytochrome c levels in cells grown under oxygen deprivation. It is therefore tempting to speculate that TRAIL resistance could be attributable to hypoxia-induced mitophagy rather than inefficient mitochondrial translocation of the pore forming Bax/Bak complex. Assigning hypoxia-induced TRAIL resistance to mitophagy is supported by our data for the following reasons: First of all, the decrease of mitochondrial mass under low oxygen levels (Figure 3B) was associated with enhanced autophagosome formation (Figure 3D). Secondly, the autophagy inhibitor 3MA reduced the loss of mitochondria under hypoxia (Figure 3E). And finally, the autophagy inhibitor 3MA reversed TRAIL resistance in hypoxic DLD1, HCT-8 and HCT116 cells (Figure 3F).

Admittedly, blocking autophagy under hypoxia using 3MA could also modulate TRAIL susceptibility by mitochondria-independent mechanisms. At least under normoxia, TRAIL can trigger a protective autophagic response that does not involve mitochondria [33, 34]. However, hypoxia-induced mitophagy depends on HIF1α [18] while TRAIL triggered autophagy does not [33]. The HIF1α inhibitor Bay87-2243 [19] restored TRAIL sensitivity under hypoxia (Figures 3H and 3I), arguing against involvement of TRAIL-triggered protective autophagic responses in hypoxia-induced TRAIL resistance.

When apoptosis is blocked, the TRAIL death receptors and CD95 can elicit pro-inflammatory signaling pathways [35, 36] with tumor promoting activities [7]. *In vivo*, CD95 activation in hypoxic metastasized colorectal cancer cells enhanced proliferation and promoted a highly invasive phenotype [37]. Consequently, ensuring or restoring full-blown cell death induction is a prerequisite for therapeutic exploitation of death receptors in cancer therapy.

Our study highlights potential new strategies to overcome hypoxia-induced TRAIL resistance, either by targeting the tumor environment (oxygen levels) or by antagonizing cellular adaptions to hypoxia (loss of mitochondria). Obviously, oxygen levels determine the degree of hypoxia-granted TRAIL resistance (Figure 1J), which is reversible upon re-oxygenation (Figure 1I). The efficacy of TRAIL-based anti-cancer therapies could therefore be boosted by increasing oxygen levels in tumors. Respiratory hyperoxia diminished hypoxia in the tumor microenvironment in a mouse tumor model [38] and additionally enhanced T- and NK-cell mediated immunological anti-tumor responses [39]. However, systemic hyperoxygenation may be technically challenging in the clinical setting. Increasing efficiency of oxygen delivery instead by promoting tumor vascularization is therefore an interesting alternative. A recent study reported that increasing tumor angiogenesis improved drug delivery, reduced tumor growth as well as metastasis and extended survival [40]. Therefore, combinatorial treatment with TRAIL and drugs promoting tumor angiogenesis might constitute a reasonable therapeutic approach.

Identification of hypoxia-induced mitophagy as the molecular mechanism underlying TRAIL resistance under oxygen deprivation allowed us to additionally characterize cancer cell intrinsic therapeutic targets. Imitating mitochondria-dependent amplification of the TRAIL-triggered death signal by exogenously adding SMAC mimetics (Figure 4C-4H) or enforcing a mitochondria-independent type-I mode of cell death by targeting XIAP, the gatekeeper of the type-II phenotype (Figure 5C-5H) [21, 22], boosted TRAIL- and CD95L-induced cell death. Both approaches also robustly killed colorectal cancer cells that exhibited high-level TRAIL resistance in the presence of ample oxygen (Figure 7). Moreover, BV6 restored cisPt- and STS-induced cell death under hypoxic conditions (Figure 8), altogether broadening the clinical applicability. SMAC mimetics do not exhibit an exclusive specificity for a single IAP protein and target to a varying degree cIAP1, cIAP2 and XIAP [41]. XIAP seems to hold a crucial role in hypoxia-induced TRAIL resistance, as the latter is tremendously reduced in genetically XIAP-deficient cells and when XIAP transcription is blocked (Figure 5). Additionally, XIAP is the most potent caspase inhibitor among the IAP proteins [42] and therefore in all probability responsible for the TRAIL resistant phenotype under hypoxia. However, we can not formally exclude an additional role for cIAP1 and cIAP2. Notably, hypoxia reduced the total cellular content of XIAP and other anti-apoptotic molecules (Figure 2A), but apparently not below the critical threshold to allow for TRAIL sensitization in the absence of mitochondria-derived apoptotic signals (Figure 1). Although TRAIL death receptors are capable to trigger caspase-dependent apoptosis and caspase-independent necroptosis [35, 43], zVAD-fmk-mediated rescue (Figure 4I and 5I) and PS exposure on the outer leaflet of the plasma membrane pointed to apoptotic cell death (Figure 4J and 5J). Killing cancer cells via apoptosis rather than necroptosis could therapeutically be desirable as the later is capable to initiate a strong inflammatory response [44].

**Figure 9:**
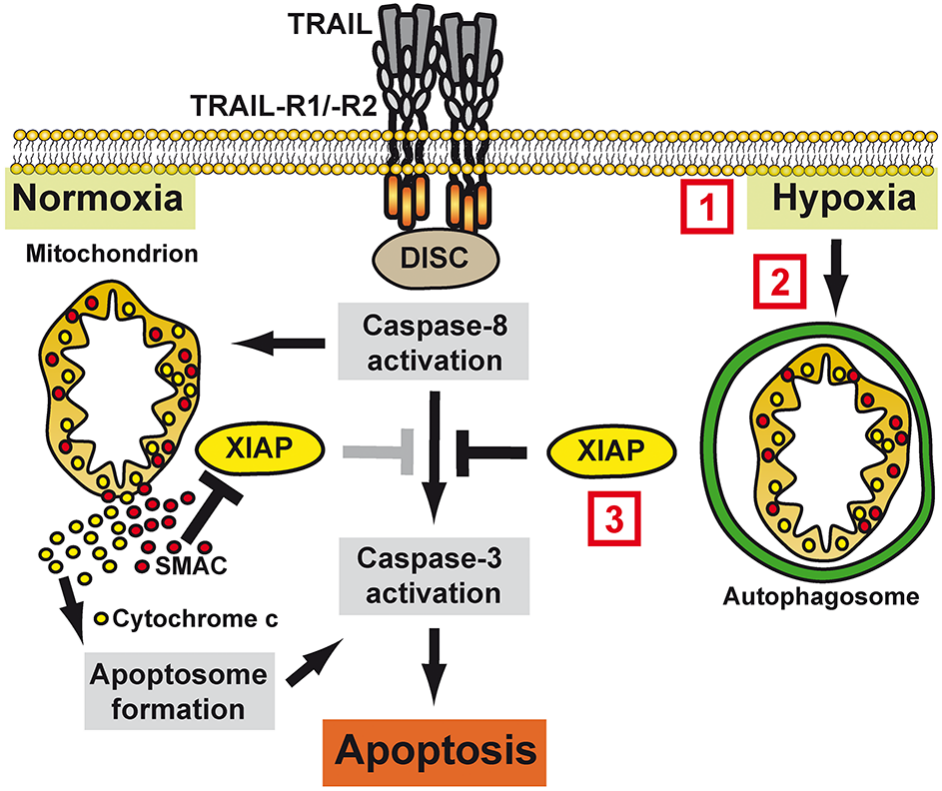
Molecular mechanism of hypoxia-induced TRAIL resistance in colorectal cancer cells Under normoxic conditions, TRAIL-R1/−R2 activation triggers DISC assembly with subsequent caspase-8 activation. Mitochondria amplify the death signal via the release of pro-apoptotic factors such as cytochrome c and SMAC, which boost caspase-3 activation by initiating apoptosome formation and antagonizing XIAP, respectively. Under hypoxic conditions, mitophagy sequesters mitochondria-derived pro-apoptotic molecules, thereby blocking efficient apoptosis induction. Therapeutically, hypoxia-induced TRAIL resistance can be overcome by **(1)** increasing local oxygen levels (e.g. systemic hyperoxia or promoting angiogenesis), **(2)** inhibition of hypoxia-induced mitophagy (e.g. Bay87-2243 targeting HIF1α or 3MA blocking autophagosome formation) or **(3)** targeting XIAP (e.g. SMAC mimetics or blocking XIAP transcription using MithA).

Taken together, we identified hypoxia-induced mitophagy as a novel mechanism to regulate TRAIL sensitivity in oxygen-deprived colorectal cancer cells and characterized cancer cell intrinsic and extrinsic approaches for re-sensitization to TRAIL-based anti-cancer therapy (summarized in Figure 9). Therapeutically, we provide experimental evidence that combinatorial treatment strategies with TRAIL and SMAC mimetics or XIAP-targeting drugs overcome the apoptosis-hampering conditions imposed by the hypoxic tumor microenvironment. Our findings extend previous approaches to overcome hypoxia-induced apoptosis resistance [29, 30] and also have a broad clinical applicability in colorectal cancer cells exhibiting TRAIL-resistance under normoxia.

## MATERIALS AND METHODS

### Cell lines, antibodies and reagents

HCT116 and DLD1 cells were purchased from the German Collection of Microorganisms and Cell Culture (DSMZ, Braunschweig, Germany). HCT-8 cells were obtained from Cell Line Service GmbH (CLS, Eppelheim, Germany). Cell lines were authenticated by STR profiling. HCT116 Bax^−/−^ [45] and HCT116 XIAP^−/−^ [46] cells were kindly provided by Bert Vogelstein (Johns Hopkins University, Baltimore, MA, USA) and Philipp Jost (III. Medizinische Klinik, Technische Universität München, Germany), respectively. Cells were cultured in RPMI 1640 medium (PAN Biotech, Aidenbach, Germany) supplemented with 10% [v/v] fetal calf serum (Sigma, Steinheim, Germany). Oxygen levels were modulated using a Whitley H35 Hypoxystation (Don Whitley Scientific, Shipley, UK). Antibodies specific for FADD, caspase-8, Bax and XIAP were purchased from Cell Signaling Technology (Beverly, MA, USA). Anti-TRAIL-R1, anti-TRAIL-R2, anti-TRAIL-R3 and anti-TRAIL-R4 antibodies were from ProSci (Poway, CA, USA), anti-HIF1α was from Cayman Chemical (Ann Arbor, MI, USA) and anti-tubulin from Dunnlab (Asbach, Germany). BV6, LCL161, Bay87-2243, staurosporine (STS), cis-platin (cisPt) and 3-methyladenine (3MA) were obtained from Selleck Chemicals (Houston, TX, USA). Mithramycin-A was from AppliChem (Darmstadt, Germany), zVAD-fmk from Bachem (Heidelberg, Germany) and MTT (3-[4,5-dimethylthiazol-2-yl]-2,5-diphenyltetrazolium bromide) from Biomol (Hamburg, Germany). Human recombinant TRAIL was purchased from Apronex (Jesenice u Prahy, Czech Republic) and CD95L was from Adipogen (Liestal, Switzerland). Fc-FLAG-scTRAIL was generated by in-frame insertion of FLAG-scTRAIL [47] into a pCR3 variant encoding the IgG1 Fc domain and a linker (kind gift from P. Schneider, Department of Biochemistry, University of Lausanne). Recombinant Fc-FLAG-TRAIL was produced in HEK293 cells as previously described for TL1A [48].

### Western blot analysis

Western blot analysis were performed essentially as described previously [7]. Briefly, cells were harvested, spun down, and dissolved in 4x Laemmli sample buffer (8% [w/v] SDS, 0.1 M dithiothreitol, 40% [v/v] glycerol, 0.2 M Tris, pH 8.0) supplemented with phosphatase inhibitor cocktails-I and -II (Sigma). Samples were sonificated, subsequently boiled (96°C, 5 min). Proteins were separated by SDS-PAGE and transferred to PVDF membranes. Incubation in TBS containing 0.1% (v/v) Tween 20 and 5% (w/v) dry milk blocked non-specific binding sites. Membranes were incubated with primary antibodies of the specificity of interest. Antigen-antibody complexes were visualized using horseradish peroxidase-conjugated secondary antibodies (Dako, Hamburg, Germany) and ECL technology (Pierce, Rockford, IL, USA). Mitochondria were isolated using the “Mitochondria Isolation Kit for Cultured Cells” (ThermoFisher Scientific, Waltham, MA, USA) following manufacturer's instructions.

### Coimmunoprecipitation

Immunoprecipitation of Fc-FLAG-scTRAIL was essentially performed as described previously for CD95L [7]. In brief, four confluent 150 cm^2^ tissue culture dishes per condition were stimulated with 1 μg/mL Fc-FLAG-scTRAIL for 90 min at 37°C, washed in ice-cold TBS, transferred in 2 mL lysis buffer (50 mM Tris–HCl, pH 7.5, 150 mM NaCl, 0.5% [v/v] NP40) supplemented with complete protease inhibitor cocktail (Roche, Mannheim, Germany), and incubated for 60 min on ice. Lysates were cleared by centrifugation (2 × 20 min, 14.000 g) and TRAIL-R1/−R2 complexes were precipitated using protein G agarose (Roche, 40 μL of a 50% [v/v] slurry) at 4°C overnight. Lysates from unstimulated cells supplemented with 50 ng/mL of Fc-FLAG-scTRAIL before adding protein G agarose served as negative control. After washing three times in TBS, agarose-bound proteins were eluted by incubation at 95°C for 10 min in 2x Laemmli sample buffer.

### Cell viability assay

Cells were seeded in 96-well plates (2×10^4^ per well) and grown in the presence of the indicated oxygen levels for 18 h. Subsequently, cells were stimulated with the indicated concentrations of the ligands in triplicates. Cell viability was determined 18 h after stimulation using 3-[4,5-dimethylthiazol-2-yl]-2,5-diphenyl tetrazolium bromide (MTT) or crystal violet staining.

### Flow cytometry

Cells were grown under normoxic (21% O_2_) or hypoxic (0.5% O_2_) conditions for 18 h, harvested, and incubated for 30 min on ice with PE-conjugated antibodies specific for the indicated TRAIL-receptors (Diaclone SAS, Besancon, France) or an appropriate isotype control (R&D Systems). Analyses were performed using FACSCanto II (BD Biosciences, Heidelberg, Germany) following standard procedures.

### Caspase activity assay and phosphatidylserine measurements

3×10^4^ cells per well were seeded in black 96-well plates (clear bottom) and grown under normoxic (21% O_2_) or hypoxic (0.5% O_2_) conditions for 18 h. Subsequently, cells were challenged with the indicated concentrations of TRAIL for 3 h (phosphatidylserine measurements), 4 h (caspase-8 measurements) or 5 h (caspase-3 measurements). Translocation of phosphatidylserine to the outer leaflet of the plasma membrane was determined using a fluorogenic Annexin V derivate (Phosphatidylserine apoptosis assay, AAT Bioquest, Sunnyvale, CA, USA). Caspase activity was determined using the fluorogenic substrates (DEVD)_2_-R110 (Caspase-3 activity kit, AAT Bioquest) or (Ac-IETD)_2_-R110 (Caspase-8 activity kit, AAT Bioquest) according to the manufacturer's instructions. Light emission was quantified using a Victor3 multilabel reader (Perkin Elmer, Waltham, MA, USA). All groups were analyzed in triplicates.

### Fluorescence-based measurements of mitochondrial mass

DLD1 cells were seeded in black 96-well plates (3×10^4^ cells/well) with clear bottom and grown under normoxic or hypoxic conditions for 18 h. Before staining, cells were washed once with 37°C warm Hank's buffered salt solution (HBSS, Sigma) and incubated with 200 nM MitoTracker Green FM (ThermoFisher Scientific) for 45 min at 37°C protected from light. Subsequently, staining solution was removed by aspiration, cells were again washed in HBSS and fluorescence intensity was measured using a Victor3 multilabel reader (Perkin Elmer, Waltham, MA, USA). All groups were analyzed in triplicates.

### Fluorescence-based autophagy assay

Autophagosome formation was measured using the “Cell Meter™ Autophagy Fluorescence Imaging Kit” (AAT Bioquest) according to manufacturer's instructions. In brief, 3×10^4^ cells per well cells were seeded in black 96-well plates and incubated for 18 h under normoxic or hypoxic conditions. Autophagosomes were stained after media removal by incubation for 1 h in PhagyGreen™ staining solution at 37°C protected from light. Subsequently, cells were washed three times and fluorescence intensity was measured using a Victor3 multilabel reader (Perkin Elmer). All groups were analyzed in triplicates.

### Measurement of cytosolic Ca^2+^ levels

Cytosolic Ca^2+^ levels were measured using the “Fluo-4 Direct Calcium Assay” (ThermoFisher Scientific). Cells were seeded in black 96-well plates (3×10^4^ per well) and incubated for 18 h under normoxic or hypoxic conditions. Subsequently, cells were challenged with TRAIL (256 ng/mL) for the indicated periods of time followed by direct addition of the Fluo-4-containing staining solution into the culture media. Fluorescence intensity was measured after 1 h incubation (37°C, protected from light) using a Victor3 multilabel reader (Perkin Elmer).

### Proteomic profiling

Oxygen-dependent changes in the relative expression of pro- and anti-apoptotic proteins were analyzed in DLD1 cells grown under normoxic (21% O_2_) or hypoxic (0.5% O_2_) conditions for 18 h using the “Human Apoptosis Antibody Array Kit” (R&D Systems, Wiesbaden, Germany) according to manufacturer's instructions. Intensity of the protein spots were quantified by densitometry using the open source software ImageJ 1.47v (Wayne Rasband; National Institutes of Health, Bethesda, MD, USA) and normalized to reference spots included in each panel.
